# A CE-ICP-MS/MS method for the determination of superparamagnetic iron oxide nanoparticles under simulated physiological conditions

**DOI:** 10.1007/s00216-020-02948-3

**Published:** 2020-09-23

**Authors:** Joanna Kruszewska, Jacek Sikorski, Jan Samsonowicz-Górski, Magdalena Matczuk

**Affiliations:** Chair of Analytical Chemistry, Faculty of Chemistry, Warsaw University of Technology, Noakowskiego St. 3, 00-664 Warsaw, Poland

**Keywords:** Capillary electrophoresis, Superparamagnetic iron oxide nanoparticles, Inductively coupled plasma tandem mass spectrometry, Human serum albumin

## Abstract

Over the past few years, superparamagnetic iron oxide nanoparticles (SPIONs) have attracted much attention due to their medicinally attractive properties and their possible application in cancer diagnosis and therapy. However, there is still a lack of appropriate methods to enable quantitative monitoring of the particle changes in a physiological environment, which could be beneficial for evaluating their in vitro and in vivo behavior. For this reason, the main goal of this study was the development of a novel capillary electrophoresis-inductively coupled plasma mass spectrometry (CE-ICP-MS/MS) method for the determination of SPIONs suitable for the future examination of their changes upon incubation with proteins under simulated physiological conditions. The type and flow rate of the collision/reaction gas were chosen with the aim of simultaneous monitoring of Fe and S. The type and concentration of the background electrolyte, applied voltage, and sample loading were optimized to obtain SPION signals of the highest intensity and minimum half-width of the peak. Analytical parameters were at a satisfactory level: reproducibility (intra- and inter-day) of migration times and peak areas (presented as RSD) in the range of 0.23–4.98%, recovery: 96.7% and 93.3%, the limit of detection (for monitoring ^56^Fe^16^O^+^ by mass-shift approach) 54 ng mL^−1^ Fe (0.97 μM) and 101 ng mL^−1^ Fe (1.82 μM) for SPIONs with carboxyl and amino terminal groups, respectively. To the best of our knowledge, this is the first reported use of CE-ICP-MS/MS for the quantification of SPIONs and monitoring of interactions with proteins.

**Electronic supplementary material:**

The online version of this article (10.1007/s00216-020-02948-3) contains supplementary material, which is available to authorized users.

## Introduction

Superparamagnetic iron oxide nanoparticles (SPIONs) are the group of nanomaterials composed of ferromagnetic compounds, such as magnetite (Fe_3_O_4_), which at a size smaller than ca. 30 nm exhibits a unique form of magnetism, i.e., superparamagnetism [1, 2]. The range of possible biomedical applications of SPIONs is wide, including magnetic resonance imaging, drug and gene delivery, magnetic hyperthermia therapy, photodynamic therapy, phototherapy, and chemotherapy [3, 4]. They can be applied as theranostic nanoprobes, having both therapeutic and diagnostic properties. So far, many iron oxide nanoparticles (NPs) have been subjected to preclinical and clinical trials, mainly as imaging agents, and some of these have already been introduced in the clinical setting [1].

The significant interest in the medical applications of SPIONs raised the need for a thorough examination of their behavior in a physiological environment. It should be noted that after entering the human bloodstream, NPs may interact with plasma proteins, creating a shell called a protein corona, which has a tremendous effect on the inherent physicochemical properties of NPs, their recognition by the immune system, and nanoparticle–cell interactions [5–7]. Therefore, accurate quantification methods which can be applied to study the plasma protein binding kinetics in vitro or in vivo and the pharmacokinetic parameters of nanomaterials in vivo are essential for designing safe and effective NP-based theranostic probes. One group of techniques enabling in vivo/in vitro quantification of SPIONs are noninvasive, imaging-based techniques, including optical imaging (fluorescence), magnetic resonance imaging, or positron emission tomography. However, they require the introduction of the fluorophore to the NP structure or specialized equipment not commonly available in analytical laboratories [8]. Another group of techniques may involve NP dissolution, like inductively coupled plasma-optical emission spectroscopy and inductively coupled plasma mass spectrometry (ICP-MS) or spectrophotometric methods [9]. On the other hand, ICP-MS used in single-particle (SP-ICP-MS) mode might be better suited for NP quantification without the need for dissolution, as it requires only a proper sample dilution [10]. A technique that could find application in SPION quantification in biological samples, having the advantage of not being affected by endogenous iron from tissues or body fluids, is magnetic susceptibility measurements [11]. Coupling of separation techniques to ICP-MS is a beneficial approach for quantification and separation of SPIONs and Fe-containing species originating from NPs. HPLC-ICP-MS has been utilized for the determination of the metabolic products of iron oxide NPs in cells and in simulated gastric acid solubility studies in vitro [12, 13]. The main obstacles to the use of the abovementioned techniques in the quantification of SPIONs and tracking their changes in biological media are the time-consuming sample preparation [9], insufficient detection limits [11], or the use of surfactant in the separation system that can influence the equilibrium of the interaction of SPIONs with biological molecules [12].

When considering Fe quantification (when measuring the most abundant isotope ^56^Fe) by ICP-MS, polyatomic interferences from precursors from several sources, including the sample matrix (^40^Ca^16^O^+^), plasma gases (^40^Ar^16^O^+^), or reagents used for preparation, need to be taken into account [14, 15]. These interferences can be reduced using sector-field ICP-MS. This type of instrument is equipped with a double-focusing sector field mass spectrometer that can be operated at a higher mass resolution than the more common quadrupole mass analyzer that only provides unit mass resolution. Another approach enabling a reduction of interferences is a collision/reaction cell (CRC) in a quadrupole-based ICP-MS instrument [16]. The CRC is an extra cell (containing a quadrupole, hexapole, or octupole) that can be pressurized with gas to remove interferences. This can be achieved by either (i) collisions of the ions with a non-reactive gas (e.g., He), in combination with kinetic energy discrimination, or (ii) selective reaction of the analyte and/or interfering ion(s) with a reactive gas (e.g., O_2_, H_2_, NH_3_). Recently developed technology based on an ICP-tandem mass spectrometer (MS/MS) introduces an additional quadrupole in front of the CRC. It can operate as a mass filter allowing only ions with a certain m/z ratio to enter the CRC. In this way, matrix ions with an m/z ratio other than the element of interest are excluded from the cell, which allows better control over the reactions taking place in the cell. ICP-MS/MS with a reactive gas enables the decrease of spectral overlap by two different approaches. The on-mass mode is based on the conversion of the interfering ion into a new one of different m/z ratio than the analyte, whereas in mass-shift mode the analyte ion reacts with the gas molecule, giving a product ion which can be determined at an m/z ratio other than that of the original analyte ion, free from the interference [17, 18].

To date, several reports on the determination of Fe by ICP-MS with CRC or ICP-MS/MS have been published. Different collision/reaction gas chemistries have been utilized for minimizing the interferences leading to a decreased background of Fe level. For example, hydrogen was used in MS/MS mode to reduce interferences during the investigation of iron NPs and their interactions with Cd^2+^ in wastewater [15] and as a reaction gas for determination of Fe_3_O_4_ NPs spiked into blood plasma by SP-ICP-MS/MS [19]. In another work, in addition to hydrogen, ammonia was used to measure Fe_3_O_4_ NPs by the same technique [10]. Ammonia reaction gas was also applied in a CRC in the capillary electrophoresis (CE)-ICP-MS method for speciation analysis of Fe(II)/(III) in biological fluids and cells [20]. Further, helium was chosen as a collision gas for HPLC-ICP-MS quantification of the iron-carbohydrate NP-based drugs in clinical samples [21]. Also, oxygen was used in a CRC for the simultaneous measurement of Fe, Zn, S, and P in cereal products [22].

Due to advantages such as low sample consumption, short analysis time, no stationary phase that could interact with the analyte, and higher resolution, CE could be a favorable alternative to HPLC in the area of SPION separation and investigation of SPION–protein interactions. Up to now, CE has been applied for the examination of SPIONs in combination with UV-Vis detection and has focused on the separation of NPs with different types of polymeric coating, different surface charges, or different sized NPs and the determination of their electrophoretic mobility. However, the quantification of NPs was not conducted in those works [23–26]. CE with a diode-array detector (DAD) was also utilized for monitoring interactions of Fe_3_O_4_ NPs with bovine serum albumin [27]. In a recent report, CE-ICP-MS/MS was employed to study interactions between carboxylated core–shell magnetic nanoparticles and antibiotic (polymyxin B) in affinity capillary electrophoresis (ACE) mode, with the aim of developing a microdevice based on SPIONs to test bacterial resistance [28]. However, the experiments were conducted under one set of electrophoretic conditions only (no optimization trials reported) and were not directed towards quantitative analysis. CE-MS hyphenation is applicable to protein-binding studies of metal-based NPs, but surprisingly has not been employed for SPIONs in serum samples so far [29].

The main aim of the present work was to develop the CE-ICP-MS/MS method allowing the separation and determination of the differently net-charged SPIONs suitable for subsequent monitoring of their speciation changes occurring upon interaction with serum proteins in the physiological environment.

## Materials and methods

### Chemicals

Superparamagnetic iron oxide nanoparticles with an amphiphilic polymer coating possessing different terminal groups [carboxyl (negative net charge) and amino (positive net charge)] were purchased from Ocean NanoTech, USA. The size of NPs, measured by bright-field scanning transmission electron microscopy (BF STEM), was 15.3 ± 2.3 nm and 20.2 ± 2.6 nm, respectively. Chemicals used to prepare the buffer solutions, i.e., Na_2_HPO_4_, NaH_2_PO_4_, 4-(2-hydroxyethyl) piperazine-1-ethanesulfonic acid (HEPES), piperazine-*N*,*N*′-bis(2-ethanesulfonic acid) (PIPES), ammonium carbonate, and ammonium bicarbonate, as well as sodium hydroxide, sodium chloride, and iron(II) chloride, were purchased from Sigma-Aldrich (St. Louis, MO, USA). Vanadium standard solution was purchased from Fluka (Buchs, Switzerland). Iron ICP standard solution was obtained from Merck Millipore (Darmstadt, Germany). Albumin from human serum was purchased from Sigma-Aldrich (USA). Ultrapure Milli-Q water was obtained from a Millipore Elix 3 system (Merck Millipore) and used throughout this study. The following gases of purity ≥99.999% (Messer, Bad Soden, Germany) were used for the collision/reaction cell in ICP-MS/MS: oxygen, helium, hydrogen, and ammonia (10% NH_3_ in He).

### Imaging of NPs by BF STEM

The size of SPIONs was established using a Hitachi SU8230 ultrahigh-resolution field emission scanning electron microscope (Hitachi High-Technologies Corporation) using BF STEM with the application of 30.0 kV accelerating voltage. The samples were imaged with the use of gold TEM grids coated with lacey carbon film. Grids were immersed in NP aqueous (water/buffer) suspensions and dried before the observation.

### Preparation of the samples

For CE-ICP-MS/MS experiments, suspensions of differently functionalized iron oxide nanoparticles were sonicated and diluted in water or 10 mM phosphate buffer, pH 7.4, containing 100 mM NaCl and incubated at physiological temperature, before analysis. Albumin from human serum was diluted in 10 mM phosphate buffer, pH 7.4, containing 100 mM NaCl. The final concentration used in the experiments was 1 mg mL^−1^, which corresponds to the average concentration of albumin in 50-fold diluted human serum. The physiological-like sample was prepared by mixing albumin from human serum (final concentration 1 mg mL^−1^) and carboxyl SPIONs (final concentration 30 μg mL^−1^) in 10 mM phosphate buffer, pH 7.4, with 100 mM NaCl and incubated at 37 °C.

So far, iron oxide NPs have been employed for example in hyperthermia therapy on mice (18 mg Fe kg^−1^ body mass) or in photothermal therapy (coated with polypyrrole) acting as a target component to guide the NPs to the tumor site under an external magnetic field (3.3 mg Fe kg^−1^ body mass) [30, 31]. The final concentration of Fe in the sample (30–50 μg mL^−1^) was determined by taking into account the abovementioned range of Fe doses and assuming the average body mass of a man as 70 kg and a blood volume of 5 L. The proper dilution of the protein-containing media also needs to be considered to prevent adsorption of the excess proteins onto the wall of the separation capillary, while also enabling observation of the sulfur signal originating from proteins. For that reason, to maintain the NP–protein ratio in the sample analogous to that in in vivo tests, the concentration of Fe in the sample cannot be too low.

### CE-ICP-MS/MS

Analyses were conducted on an Agilent 7100 CE system coupled to an Agilent 8900 ICP tandem mass spectrometer (Agilent Technologies, USA) working in MS/MS mode using collision/reaction gases. Polyimide-coated fused silica capillaries (i.d. 75 μm; o.d. 375 μm; length 70 cm) were purchased from C&M Scientific Ltd., UK. The liquid-introduction system comprised a CEI-100 nebulizer (Teledyne CETAC Technologies, USA) equipped with a low-volume spray chamber and a crosspiece to merge the sheath liquid flow. The electrical circuit was closed by a grounded platinum wire. Instrument control and data analysis were performed with the use of Agilent MassHunter software. The new capillary was activated by rinsing with 1 M NaOH for 45 min followed by purging for 10 min with water. Before each analysis, the capillary was washed with 1 M NaOH for 1.5 min, a mixture of 1 M NaOH, methanol, and water (25/50/25, *v*/v/v) for 1 min, and water for 1 min, and finally equilibrated with the background electrolyte (BGE) for 2 min. Such a procedure enabled the elimination of memory effect and ensured long capillary lifetime. Samples were introduced via hydrodynamic injection for 5 s with 50 mbar pressure.

To calculate the recovery, internal pressure (20 mbar) was applied during the electrophoretic run. Then, the sum of the peak areas obtained in the optimized conditions for the sample containing a mixture of two types of SPIONs was divided by the sum of the peak areas obtained for the analysis with the use of internal pressure. The resolution was calculated based on the equation: 2(*t*_2_ − *t*_1_)/(*W*_1_ + *W*_2_), where t_1_, t_2_, and W_1_, W_2_ are the migration times of the peaks and peak width at the base, respectively. Electrophoretic mobility values were calculated based on the migration time of acetone as a neutral analyte. The absorbance was measured by the diode-array detector (DAD) at a wavelength of 270 nm, and the obtained migration time of acetone was recalculated, considering the effective length of the capillary.

## Results and discussion

### Evaluation of oxygen as reaction gas and optimization of oxygen flow rate

Determination of iron by ICP-MS/MS is usually performed with the use of hydrogen in a CRC, which helps to minimize spectral interference (^40^Ar^16^O^+^). However, the addition of hydrogen does not overcome the problem of spectral interference in the case of sulfur [22]. In order to apply the method for biological samples containing proteins, oxygen was preselected as the reaction gas appropriate for measuring the marker of proteins - sulfur (^32^S^+^ ⟶ ^32^S^16^O^+^) and its performance for Fe determination was evaluated in comparison to other gases (for details see Electronic Supplementary Material, ESM) [32].

Vanadium was used as an internal standard in the sheath liquid for the CE-ICP-MS/MS. Monitoring the internal standard throughout the analysis and after the capillary rinsing was essential for the control of hyphenation stability and nebulization efficiency. As ^51^V^+^ can suffer from interference from ^35^Cl^16^O^+^, the ^51^V^16^O^+^ signal was measured by a mass-shift approach. The analyses were only initiated when the signal of ^51^V^+^ ⟶ ^51^V^16^O^+^ was sufficiently stable (relative standard deviation [RSD] < 2%), and high (counts per second [cps] > 8000, for 10 ng mL^−1^ vanadium).

During the tuning of the instrument, different gas flow rate values were tested to obtain the highest intensity of ^51^V^16^O^+^ signal and at the same time the lowest possible ^51^V^+^ signal using CE-ICP-MS/MS. Oxygen flow at 32% of the maximum flow value (0.48 mL min^−1^) was selected, as it ensured the highest conversion rate of ^51^V^+^. The flow rate of oxygen was also verified with the use of sheath liquid containing vanadium, iron, and sulfur. The chosen gas flow value ensured compromised conditions allowing the highest possible signals of ^51^V^16^O^+^, ^56^Fe^16^O^+^, and ^32^S^16^O^+^ measured simultaneously. To check for possible interference for on-mass and mass-shift monitoring of Fe coming from the matrix, the sample containing albumin (the protein responsible for binding of the metal ions and calcium ions, among others) in 10 mM phosphate buffer was analyzed by CE-ICP-MS/MS. No signals coming from possible transitions ^40^Ca^16^O^+^ → ^40^Ca^16^O^+^ or ^40^Ca^16^O^+^ → ^40^Ca^16^O_2_^+^ were observed. Due to the higher background for on-mass monitoring of ^56^Fe^+^ by CE-ICP-MS/MS, a mass-shift approach was chosen for further monitoring of SPIONs.

### Selection of CE separation conditions

The main aim of the optimization trials was to find the type, concentration, and pH of the BGE, and the applied voltage that would ensure the highest sum of peak areas, the best recovery of Fe from the separation capillary, and the broadest electrophoretic sample zone, which would allow separation of the substrates and products of NP–protein interactions in future experiments. For this reason, the optimization of separation conditions was conducted using the sample containing SPIONs with opposite surface charge - carboxyl and amino functionalization groups (50 μg mL^−1^ Fe) - diluted with water.

**Fig. 1:**
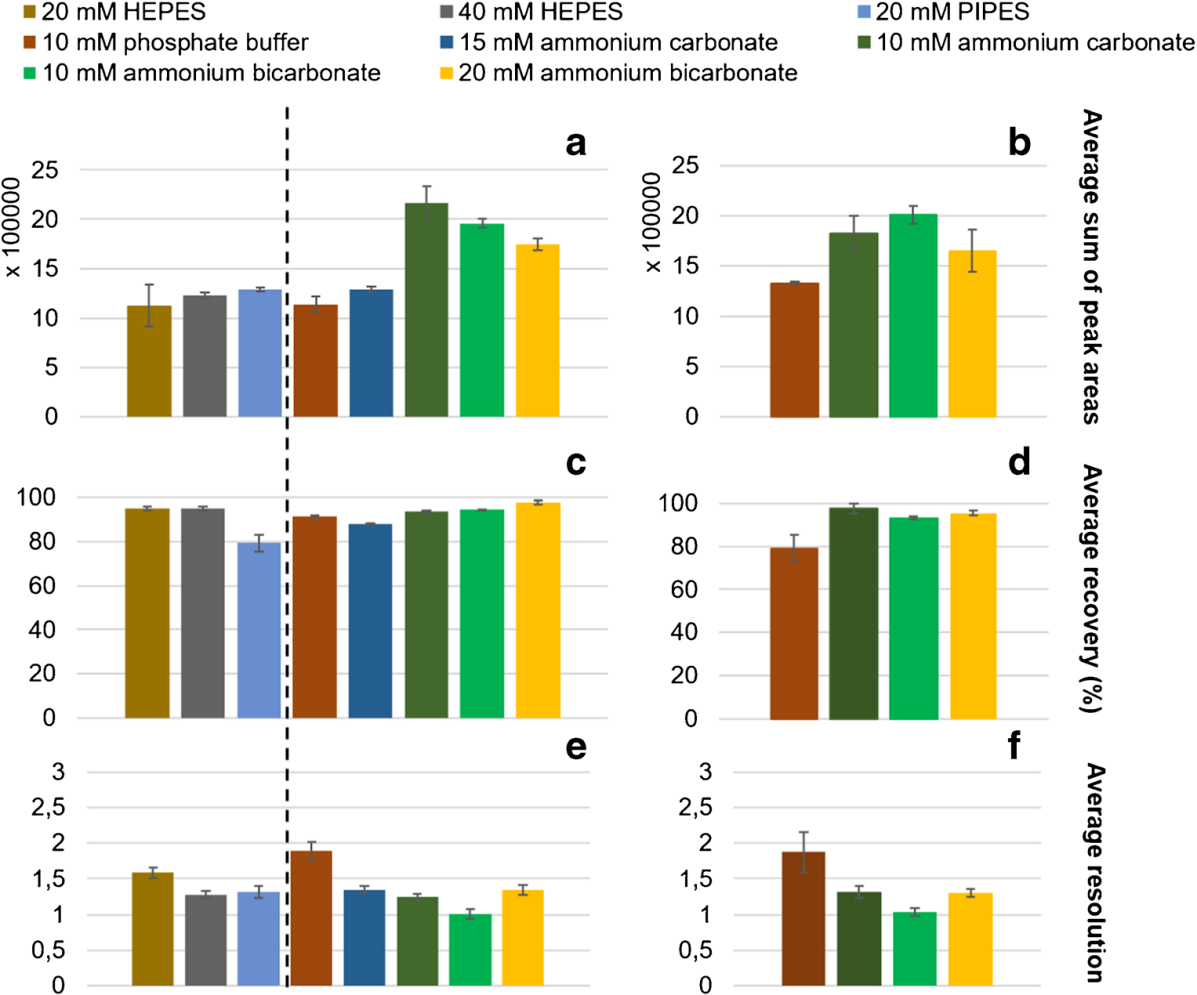
Influence of the buffer type and pH on the average sum of peak areas (**a**, **b**), recovery (**c**, **d**), and migration time difference (**e**, **f**) of SPIONs with carboxyl and amino terminal groups (50 μg mL^−1^ each); applied voltage: 18 kV, injection volume: 150 mbar × s, pH of BGE 7.4 (**a**, **c**, **e**), 8.0 (**b**, **d**, **f**), MS/MS signal ^56^Fe^16^O^+^, *n* = 6

At the beginning of optimization tests, different types of buffers, presented in Fig. 1, were tested as BGE. In order to mimic the physiological conditions of the human bloodstream, only two pH values (7.4 and 8.0) were tested. The selection of tested buffer types and concentrations was narrowed by the upper limit of the current value in the CE-ICP-MS/MS hyphenation (50 μA). Good’s buffers (HEPES and PIPES) were tested for comparison purposes; however, since sulfur is one of their components, their further application was not considered. The best results (recovery, a sum of peak areas) were obtained in the case of ammonium bicarbonate and ammonium carbonate buffers. The influence of BGE pH was not significant, so physiological 7.4 was chosen for the subsequent tests. The highest sum of peak area can be ascribed to the good volatility of the abovementioned BGEs, which promotes the ionization of the analytes. Ammonium bicarbonate (20 mM), pH 7.4, was chosen for further experiments, due to the satisfactory average resolution (Fig. 1e). Also, this type of buffer provided the highest capillary recovery values (Fig. 1c). The influence of the BGE type on separation effectiveness is presented in ESM Fig. S1. The shoulder peak (1) on electropherogram A is caused by the unstable aspiration of the sheath liquid for that moment of analysis. Although 10 mM phosphate buffer ensured the best resolution, it was eliminated from further experiments due to the broadening of the signal of SPIONs with carboxyl terminal groups (ESM Fig. S1). Also, the sum of peak area obtained for phosphate buffer as BGE was poor, probably because of the possible precipitation of phosphates in the CE-ICP-MS interface.

**Fig. 2:**
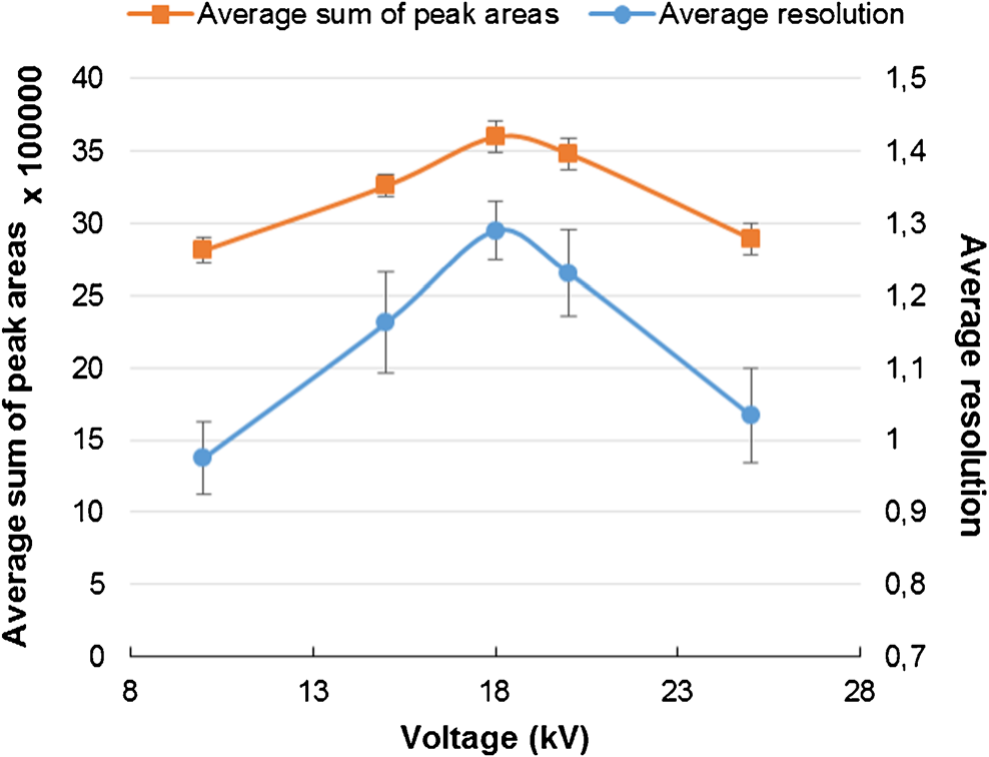
Effect of applied voltage on the average sum of peak areas and average resolution of Fe for the mixture of carboxyl and amino SPIONs (50 μg mL^−1^ each), BGE: 20 mM ammonium bicarbonate, pH 7.4, injection volume: 250 mbar × s, MS/MS signal ^56^Fe^16^O^+^*, n* = 6

The impact of sample volume on the capillary recovery was also studied (for optimal BGE; the rest of the parameters were not changed). The highest tested sample volume (250 mbar × s) was chosen because these conditions did not cause the recovery decrease. Next, the influence of applied voltage on the resolution of separation and sum of peak areas was studied for 20 mM of ammonium bicarbonate, pH 7.4 (Fig. 2), and sample volume 250 mbar × s. Eighteen kilovolts was chosen as an optimal voltage value due to the highest sum of peak area and best resolution. For lower voltage values, the peak width was higher, which resulted in worse resolution. The optimized parameters of the CE-ICP-MS/MS method are summarized in Table 1.

**Table 1:** Optimal CE-ICP-MS/MS parameters

CE system	
BGE	Ammonium bicarbonate, 20 mM, pH 7.4
Voltage	+18 kV
Temperature	37 °C
Current	26–27 μA
ICP-MS/MS system	
RF power	1550 W
Sample depth	8.0 mm
Nebulizer gas flow	1.00 L min^−1^
Cell gas (O_2_) flow	0.48 mL min^−1^
Monitored isotopes	^56^Fe^+^ → ^56^Fe^+^, ^56^Fe^+^ → ^56^Fe^16^O^+^, ^51^V^+^ → ^51^V^16^O^+^, ^32^S^+^ → ^32^S^16^O^+^

### Analytical parameters of the optimized CE-ICP-MS/MS assay

The repeatability and reproducibility of migration time and peak area were tested for two types of SPIONs. The samples containing amino or carboxyl SPIONs (50 μg mL^−1^) were analyzed six times every day for 3 days, and the results are presented in Table 2 as the relative standard deviation (RSD). The precision of the method was found to be better in terms of migration time than peak area, being below 1% for intraday and below 3.5% for inter-day measurements. A slightly lower recovery was noted for amino SPIONs in comparison to carboxyl SPIONs, which indicates that the influence of the type of terminal group on SPION interactions with the inner wall of the separation capillary is not significant.

**Table 2:** Figures of merit of the optimized CE-ICP-MS/MS method

Functional group	RSD (%)				Capillary recovery (%) (*n* = 3)	Detection limit for MS/MS		Average effective electrophoretic mobility (×10^9^, m^2^ V^−1^ s^−1^, *n* = 6)
	Migration time		Peak area					
	Intraday (*n* = 6)	Inter-day (*n* = 3)	Intraday (*n* = 6)	Inter-day (*n* = 3)		^56^Fe^+^ (ng mL^−1^ Fe)	^56^Fe^16^O^+^ (ng mL^−1^ Fe)	
Carboxyl	0.23	1.92	4.27	4.98	96.7	2.9	54	−5.69 ± 0.03
Amino	0.52	3.25	2.81	4.95	93.3	5.0	101	11.41 ± 0.02

The limit of detection (LOD) calculated based on the signal-to-noise ratio was dependent on the terminal group type. This difference in LOD is associated with different efficiency of vaporization and ionization of carboxyl and amino SPIONs. LOD values for on-mass mode (^56^Fe^+^ → ^56^Fe^+^) were 2.9 ng mL^−1^ (51 nM) and 5.0 ng mL^−1^ (89 nM) for carboxyl and amino functionalization groups, respectively, while for mass-shift mode (^56^Fe^+^ → ^56^Fe^16^O^+^), the LOD values were around 20 times higher: 54 and 101 ng mL^−1^ (0.97 and 1.82 μM). Although the level of baseline was higher for Fe measurement using the on-mass approach than for mass-shift mode (mainly due to the interferences coming from ^40^Ar^16^O^+^), the signal-to-noise ratio was found to be significantly higher. The LOD values obtained for on-mass mode are at the same range of magnitude when compared with the application of the HPLC-ICP-MS technique for iron oxide nanoparticle determination [12]. However, the mass-shift approach was chosen in this work due to the lower background level. The calibration curves (range 0.5–50 μg mL^−1^) obtained for two types of particles and two gas modes are presented in ESM Fig. S2. For amino-functionalized SPIONs, the correlation coefficients were 0.9948 (on-mass mode) and 0.9929 (mass-shift mode), and for carboxyl-functionalized SPIONs they were 0.9939 (on-mass mode) and 0.9807 (mass-shift mode). Moreover, the efficiency of the optimal method (for on-mass mode) was determined and compared with the efficiency of the method where the oxygen gas was not applied to the MS/MS system (interferences not eliminated, ESM Fig. S3). Without gas, the size and shape of SPION signals were drastically worse, while the baseline was extremely high (800,000 cps). These findings led to the conclusion that the employment of a CRC is necessary for accurate determination of Fe-containing species. In comparison to a standard ICP-MS instrument with a single quadrupole, the application of tandem mass spectrometry ensures better performance of the method due to the possibility for removal of interfering ions in the first quadrupole.

### Stability of SPIONs under simulated physiological conditions

**Fig. 3:**
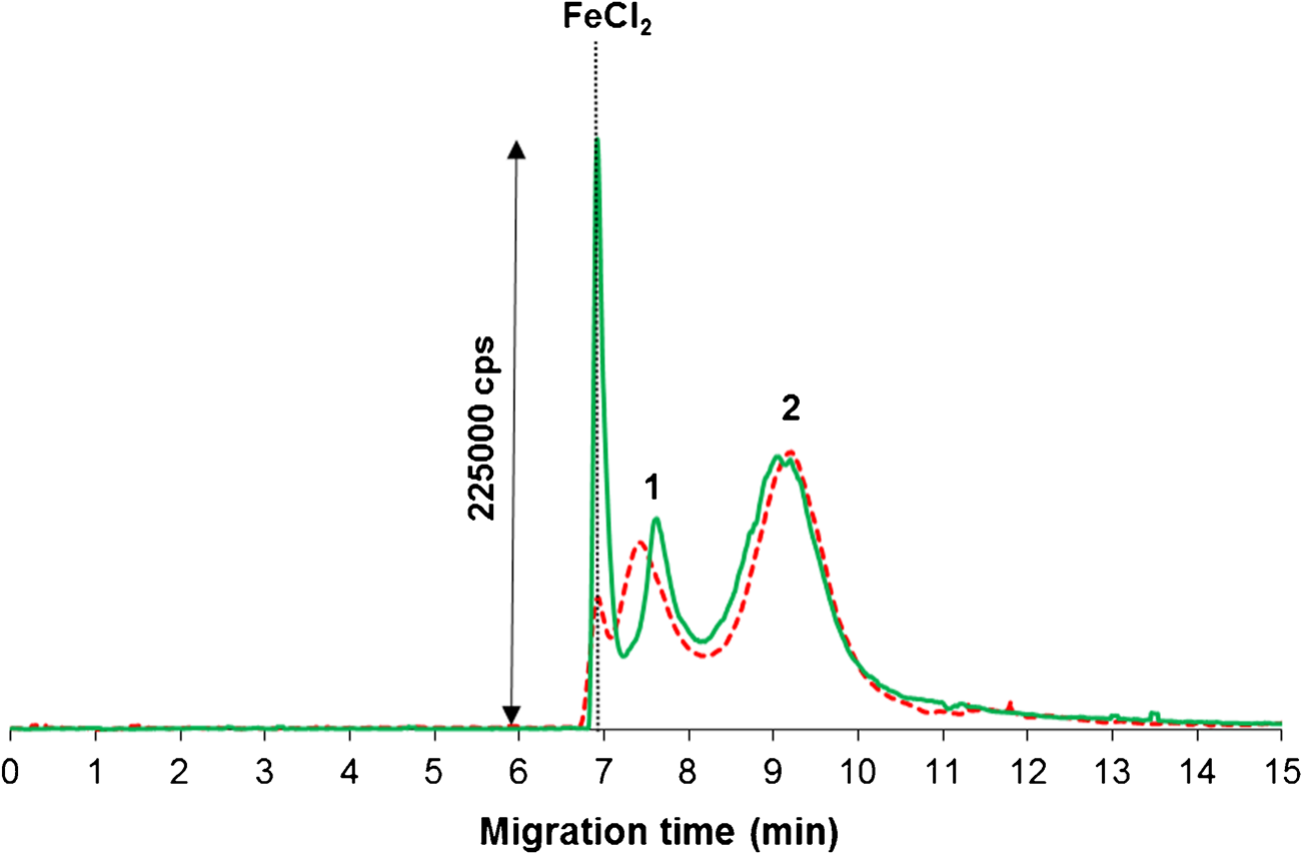
CE-ICP-MS electropherograms (MS/MS signal ^56^Fe^16^O^+^) of carboxyl (signal no. 2) and amino (signal no. 1) SPIONs with 30 μg mL^−1^ Fe suspended in 10 mM phosphate buffer, pH 7.4, and 100 mM NaCl after 3 h (red dotted line) and 24 h (green line) of incubation at 37 °C; migration time for FeCl_2_ marked as a black dotted line; separation under optimal conditions (see Table 1)

The optimized CE-ICP-MS/MS method was tested as a tool for the measurements of SPION stability under simple simulated serum conditions as a prerequisite for monitoring of SPION alterations in human serum. For this purpose, the carboxyl and amino SPIONs (30 μg mL^−1^ Fe) were diluted with 10 mM phosphate buffer, pH 7.4, and 100 mM NaCl, and the samples were analyzed 3 h and 24 h after mixing. As presented in Fig. 3, carboxyl SPIONs were stable under those conditions even after 24 h, while some of the amino SPIONs were transformed into a form with higher electrophoretic mobility. The new signal migration time value was comparable to the signal obtained for the ionic form of iron (FeCl_2_) in physiological-mimicking medium (10 mM phosphate buffer, pH 7.4, 100 mM NaCl). The signal assignment was based on the migration times of SPIONs measured for the samples containing one type of SPION only. The obtained results confirm the decomposition of amino SPION. It should be noted that after 24 h of incubation, about 40% of amino SPIONs were decomposed. The decomposition of amino SPIONs to low-molecular-weight compounds was also confirmed by the analysis of the ultrafiltrate (3-kDa pore size, 30 min, 12,000 rpm). In this case, on the electropherogram, only the signal with migration time of about 7 min was noted. The stability of SPIONs was additionally verified by BF STEM imaging. SPIONs suspended in phosphate buffer for 24 h preserved their spherical shape. Also, simulated physiological conditions did not lead to NP agglomeration (as can be seen in ESM Fig. S4). Unfortunately, it was also observed that the application of chloride in the sample medium (high ionic strength) caused the narrowing of the sample electrophoretic zone, which resulted in changes in SPION migration time and a decrease in the quality of differently charged SPION separations. For this reason, in the next step of experiments, only stable carboxyl SPIONs were selected.

### Monitoring of carboxyl SPION changes after interaction with human serum albumin

**Fig. 4:**
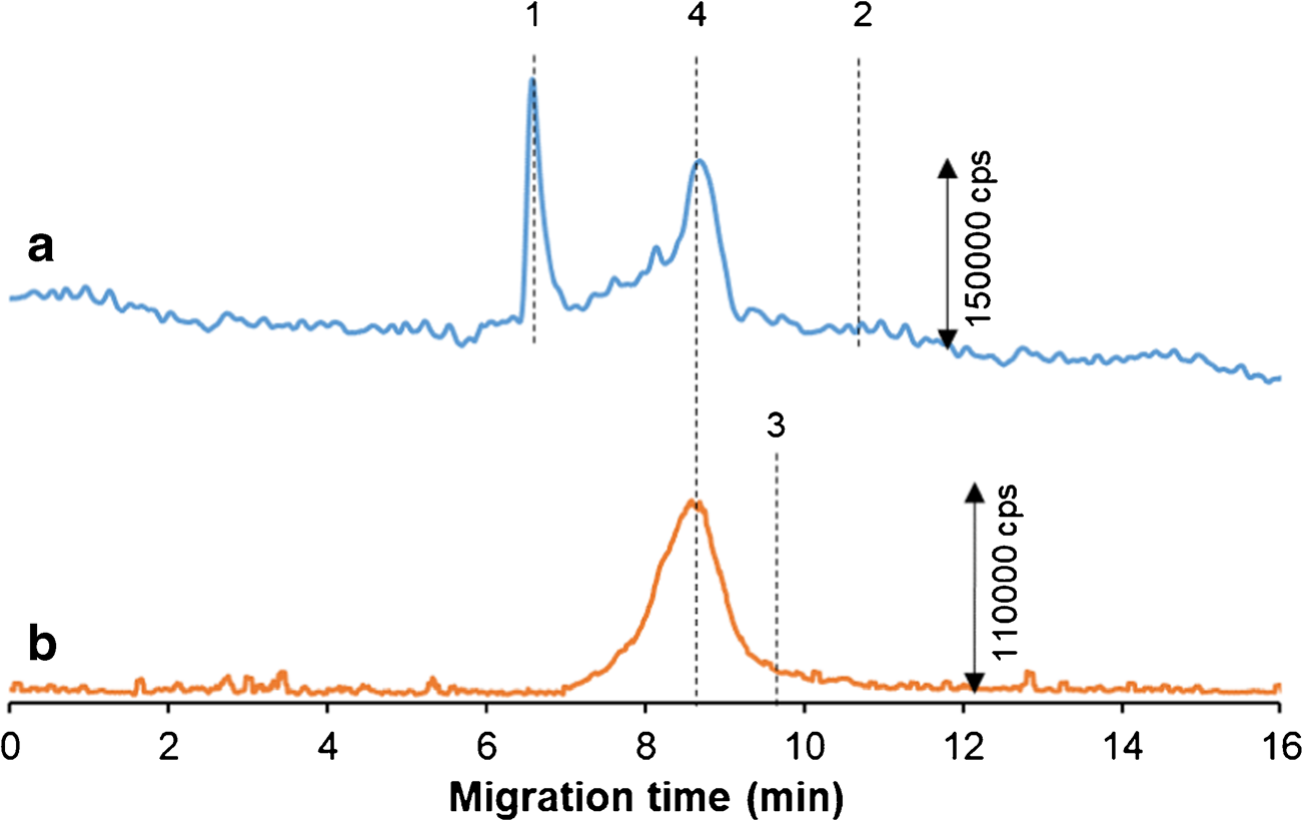
CE-ICP-MS electropherogram MS/MS signal ^32^S^16^O^+^ (**a**) and MS/MS signal ^56^Fe^16^O^+^ (**b**) of the mixture of carboxyl SPIONs (30 μg mL^−1^ Fe) and albumin (1 mg mL^−1^) diluted in 10 mM phosphate buffer, pH 7.4, and 100 mM NaCl, and incubated at 37 °C for 30 min (molar ratio of albumin and SPIONs was 4000:1). Signal assignments: blank sulfur signal (1), albumin (2), carboxyl SPIONs (3), carboxyl SPION–albumin conjugates (4); separation under optimal conditions (see Table 1)

To verify the applicability of the proposed method for investigation of SPION changes in biological samples, the mixture of carboxyl SPIONs and albumin (after 30 min of incubation at 37 °C) was tested. Previously, the migration times of reagents were established (and assigned in Fig. 4). In order to investigate the signals corresponding to protein, monitoring of the sulfur marker was carried out (^32^S^16^O^+^). It is worth mentioning that in the blank (10 mM phosphate buffer, pH 7.4, 100 mM NaCl), some sulfur signal was noted (6.5 min; signal no. 1 in Fig. 4). After 30 min of nanoparticle–protein interaction, there were no detectable peaks for albumin (Fig. 4a) or carboxyl SPIONs (Fig. 4b). This leads to the conclusion that the majority of nanoparticles and protein molecules present in the sample formed a conjugate (signal no. 4): the native (non-conjugated) SPION and albumin concentration could be below the LOD of the method. The signal corresponding to the SPIONs with a protein corona on their surface has a different migration time than polymer-coated SPIONs and consists of both sulfur and iron elements. These preliminary findings are proof that the optimized CE-ICP-MS/MS method with the application of oxygen cell gas can be used in the future for analyses of iron-based nanoparticle changes in protein-containing samples.

## Conclusions

The method presented in this work enables the separation of differently charged SPIONs by CE, in short analysis time, and their quantification by tandem ICP-MS, in a wide range of concentrations. Unlike previously described HPLC-based methods, separation is performed in mild conditions, free of organic solvents or surfactants. Moreover, the optimized method is a suitable assay for the investigation of SPION interactions with biological species. In addition to the abovementioned advantages of CE separation, the employment of ICP-MS/MS detection in oxygen mode makes this approach suitable for both sulfur and iron determination. The development and implementation of this method opens the door for novel applications of CE-ICP-MS/MS as a technique dedicated to speciation analysis of interference-problematic nano-objects in human serum.

## Electronic supplementary material

ESM 1 (PDF 651 kb)
