# Supplementary material for: A CE-ICP-MS/MS method for the determination of superparamagnetic iron oxide nanoparticles under simulated physiological conditions
Source: Anal Bioanal Chem. 2020 Sep 23;412(29):8145–53. doi: 10.1007/s00216-020-02948-3 (PMC7584539; doi:10.1007/s00216-020-02948-3)
Supplement: Supplementary file 1 — (PDF 651 kb) [file 216_2020_2948_MOESM1_ESM.pdf]

**Analytical and Bioanalytical Chemistry**

**Electronic Supplementary Material**

# A CE-ICP-MS/MS method for the determination of the superparamagnetic iron oxide nanoparticles under simulated physiological conditions

Joanna Kruszewska, Jacek Sikorski, Jan Samsonowicz-Górski, Magdalena Matczuk

**Evaluation of the performance of oxygen as a reaction gas**

In order to compare the performance of oxygen with other reaction gases for Fe determination by ICP-MS/MS, several calibration curves were prepared. Samples containing iron standard (0-50 ng $mL^{-1}$) in 2% $HNO_3$ were measured using oxygen, helium, hydrogen and ammonia. The obtained parameters of the calibration curves are presented in Table S1. Oxygen ensured the highest sensitivity (the directional coefficient of linear regression) and low limit of detection (LOD, calculated as 3 times the signal noise of the baseline) value for mass-shift determination of Fe.

The signal intensity of Fe (for on-mass and mass-shift modes) was measured for solutions containing 100 ng $mL^{-1}$ of ionic Fe and 100 ng $mL^{-1}$ of Fe in NPs to check if transport, nebulization and ionization efficiency of NPs are comparable to Fe ions. The signal intensities of Fe for NPs were 94% (on-mass mode) and 92% (mass-shift mode) of the signals noted for ionic solutions. The results confirm the assumption that transport and ionization efficiency of NPs and ions are very similar, as found in the literature, which means that the instrument can be tuned with the use of the standard of ionic Fe, not NPs [1].

**Table S1** Parameters of the analytical method for different reaction/collision gases

| Gas type | Monitored transition | Equation of calibration curve | $R^2$ value | LOD (ng $mL^{-1}$) | $LOD_{no\ gas}$/ $LOD_{with\ gas}$ |
|---|---|---|---|---|---|
| No gas | $^{56}Fe^+ \rightarrow {}^{56}Fe^+$ | y=0.07x+7.44 | 0.9779 | 5.20 | |
| Hydrogen | $^{56}Fe^+ \rightarrow {}^{56}Fe^+$ | y=0.07x+0.53 | 0.9963 | 0.17 | 30.6 |
| Helium | $^{56}Fe^+ \rightarrow {}^{56}Fe^+$ | y=0.04x+0.89 | 0.9973 | 0.10 | 52.0 |
| Oxygen | $^{56}Fe^+ \rightarrow {}^{56}Fe^+$ | y=0.06x+0.95 | 0.9966 | 0.13 | 40.0 |
| Ammonia | $^{56}Fe^+ \rightarrow {}^{56}Fe^+$ | y=0.07x+0.92 | 0.9966 | 0.08 | 65.0 |
| Ammonia | $^{56}Fe^+ \rightarrow {}^{56}Fe^{15}(NH)^+$ | y=0.35x+1.86 | 0.8416 | 2.46 | 2.1 |
| **Oxygen** | $\mathbf{^{56}Fe^+ \rightarrow {}^{56}Fe^{16}O^+}$ | **y=0.36x+1.23** | **0.9968** | **0.17** | **30.6** |
| Ammonia | $^{56}Fe^+ \rightarrow {}^{56}Fe^{16}(NH_2)^+$ | y=0.12x+0.45 | 0.9903 | 2.63 | 2.3 |
| Ammonia | $^{56}Fe^+ \rightarrow {}^{56}Fe^{17}(NH_3)^+$ | y=0.10x+0.34 | 0.9966 | 0.22 | 23.6 |
| Ammonia | $^{56}Fe^+ \rightarrow {}^{56}Fe^{18}(NH_4)^+$ | y=1.25x+6.21 | 0.9930 | 4.10 | 1.3 |
| Ammonia | $^{56}Fe^+ \rightarrow {}^{56}Fe^{16}(NH_2)_2^+$ | y=1.40x+6.77 | 0.9649 | 3.38 | 1.5 |
| Ammonia | $^{56}Fe^+ \rightarrow {}^{56}Fe^{16}(NH_3)_2^+$ | y=0.26x+0.96 | 0.9971 | 0.56 | 9.2 |

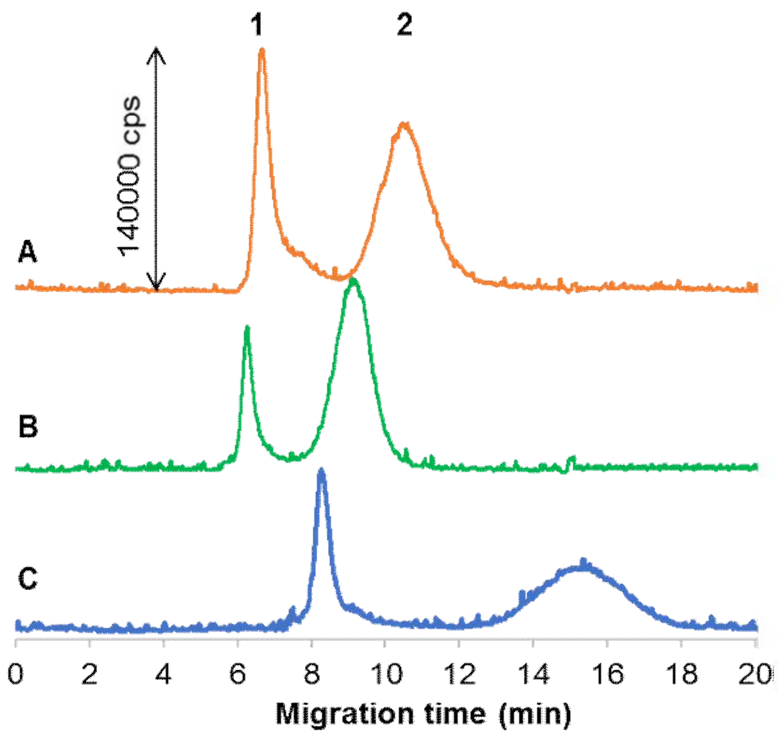


**Fig. S1** Electropherograms of 50 µg $mL^{-1}$ Fe SPIONs with (1) amino and (2) carboxy terminal groups recorded with different BGEs: (A) 20 mM ammonium bicarbonate, pH 7.4, (B) 40 mM HEPES pH 7.4, (C) 10 mM phosphate buffer, pH 7.4; applied voltage: 18 kV, injection volume: 150 mbar × s, MS/MS signal $^{56}Fe^{16}O^{+}$

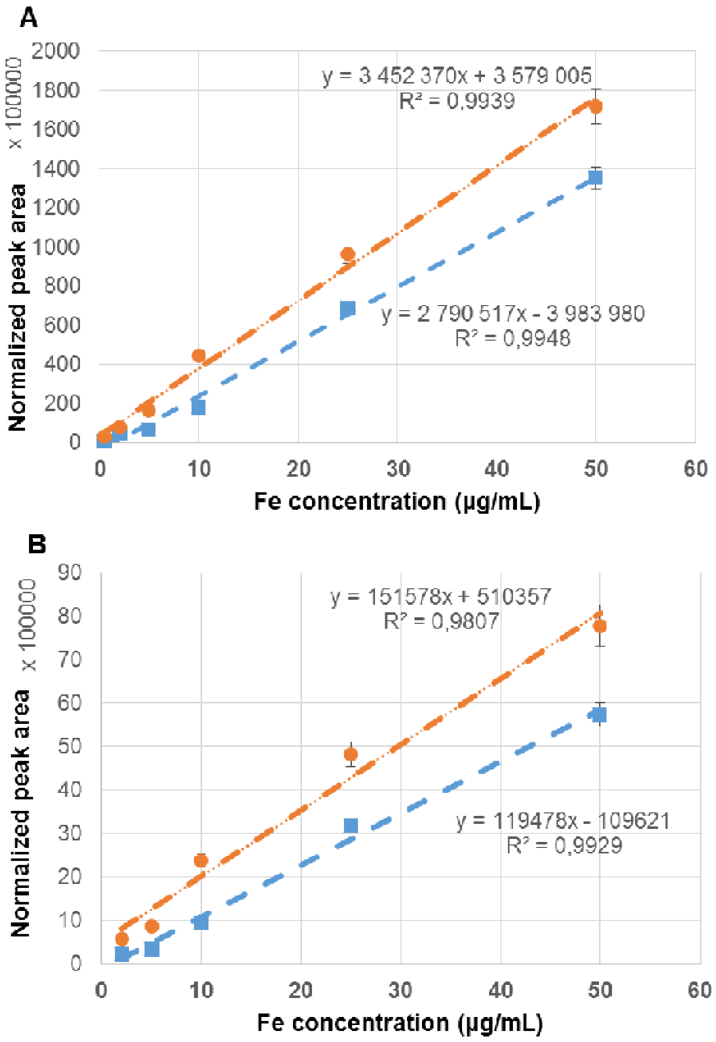


**Fig. S2** Linear range of the optimized CE-ICP-MS/MS method, (A) MS/MS signal $^{56}Fe^{+}$ (B) MS/MS signal $^{56}Fe^{16}O^{+}$, blue squares: amino SPIONs; orange circles: carboxyl SPIONs diluted in water, *n* = 6

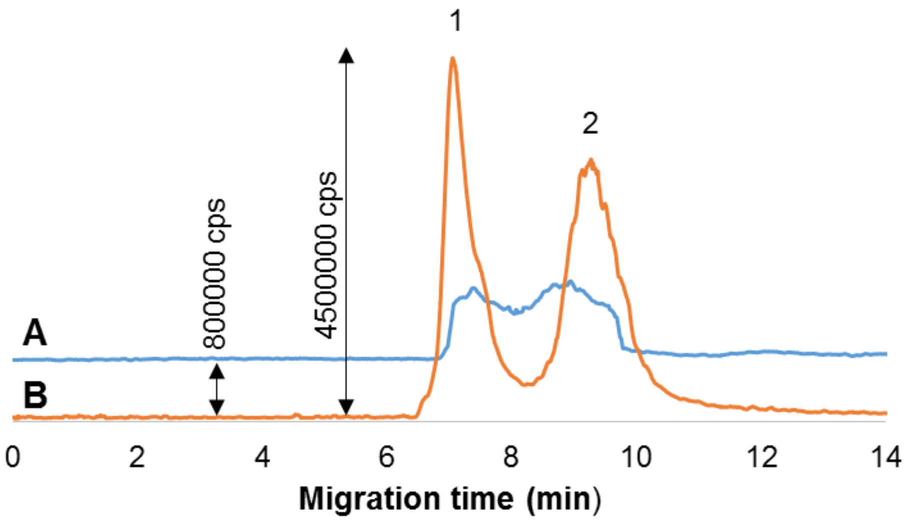


**Fig. S3** Electropherograms of 50 µg $mL^{-1}$ Fe SPIONs (MS/MS signal $^{56}Fe^{+}$) with (1) amino and (2) carboxyl terminal groups for oxygen flow rate (A) 0 and (B) 0.45 mL $min^{-1}$ (BGE: 20 mM ammonium bicarbonate, pH 7.4; applied voltage: 18 kV, injection volume: 250 mbar × s)

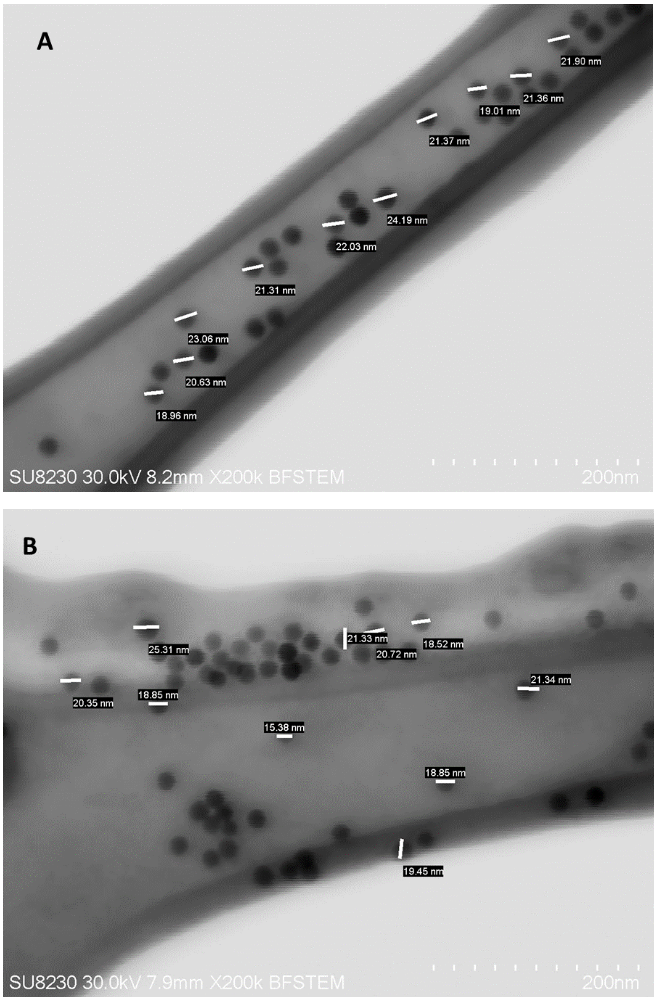


**Fig. S4** STEM photographs of SPIONs (50 µg $mL^{-1}$ Fe) with amino terminal groups 24 h after dilution in (A) water and (B) 10 mM phosphate buffer, 100 mM NaCl, pH 7.4

## References

1. Pace HE, Rogers NJ, Jarolimek C, Coleman VA, Higgins CP, Ranville JF, Determining transport efficiency for the purpose of counting and sizing nanoparticles via single particle inductively coupled plasma mass spectrometry, Anal Chem. 2012;83:9361–9369.
